# Mosaic RBD Nanoparticles Elicit Protective Immunity Against Multiple Human Coronaviruses in Animal Models

**DOI:** 10.1002/advs.202303366

**Published:** 2023-12-17

**Authors:** Yanjun Zhang, Jing Sun, Jian Zheng, Suxiang Li, Haiyue Rao, Jun Dai, Zhaoyong Zhang, Yanqun Wang, Donglan Liu, Zhao Chen, Wei Ran, Airu Zhu, Fang Li, Qihong Yan, Yiliang Wang, Kuai Yu, Shengnan Zhang, Dong Wang, Yanhong Tang, Banghui Liu, Linling Cheng, Jiandong Huo, Stanley Perlman, Jingxian Zhao, Jincun Zhao

**Affiliations:** ^1^ State Key Laboratory of Respiratory Disease, National Clinical Research Center for Respiratory Disease Guangzhou Institute of Respiratory Health the First Affiliated Hospital of Guangzhou Medical University Guangzhou 510300 P. R. China; ^2^ Department of Microbiology and Immunology University of Iowa Iowa City IA 52242 USA; ^3^ Guangzhou Customs District Technology Center Guangzhou 510700 P. R. China; ^4^ State Key Laboratory of Respiratory Disease Guangdong Laboratory of Computational Biomedicine Guangzhou Institutes of Biomedicine and Health Chinese Academy of Sciences Guangzhou 510530 P. R. China; ^5^ Guangzhou laboratory Bio‐island Guangzhou 510320 P. R. China; ^6^ Institute of Infectious disease Guangzhou Eighth People's Hospital of Guangzhou Medical University Guangzhou 510060 P. R. China; ^7^ Institute for Hepatology National Clinical Research Center for Infectious Disease Shenzhen Third People's Hospital the Second Affiliated Hospital School of Medicine Southern University of Science and Technology Shenzhen 518112 P. R. China; ^8^ Shanghai Institute for Advanced Immunochemical Studies School of Life Science and Technology ShanghaiTech University Shanghai 201210 China

**Keywords:** mosaic RBD nanoparticle vaccine coronavirus

## Abstract

To combat SARS‐CoV‐2 variants and MERS‐CoV, as well as the potential re‐emergence of SARS‐CoV and spillovers of sarbecoviruses, which pose a significant threat to global public health, vaccines that can confer broad‐spectrum protection against betacoronaviruses (β‐CoVs) are urgently needed. A mosaic ferritin nanoparticle vaccine is developed that co‐displays the spike receptor‐binding domains of SARS‐CoV, MERS‐CoV, and SARS‐CoV‐2 Wild‐type (WT) strain and evaluated its immunogenicity and protective efficacy in mice and nonhuman primates. A low dose of 10 µg administered at a 21‐day interval induced a Th1‐biased immune response in mice and elicited robust cross‐reactive neutralizing antibody responses against a variety of β‐CoVs, including a series of SARS‐CoV‐2 variants. It is also able to effectively protect against challenges of SARS‐CoV, MERS‐CoV, and SARS‐CoV‐2 variants in not only young mice but also the more vulnerable mice through induction of long‐lived immunity. Together, these results suggest that this mosaic 3‐RBD nanoparticle has the potential to be developed as a pan‐β‐CoV vaccine.

## Graphical abstract

The mosaic ferritin nanoparticle vaccine that co‐displays the spike receptor‐binding domains of SARS‐CoV, MERS‐CoV, and SARS‐CoV‐2 Wild‐type (WT) strain induce Th1‐biased immune response in mice and elicits robust cross‐reactive neutralizing antibody responses against a variety of β‐CoVs, including a series of SARS‐CoV‐2 variants. This vaccine effectively protects against challenges of SARS‐CoV, MERS‐CoV, and SARS‐CoV‐2 variants in mice.

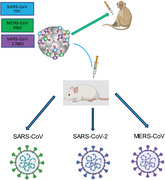

## 1 Introduction

To date, seven coronaviruses have been identified to cause disease in humans, among which human coronavirus (HCoV)‐NL63, −229E, ‐HKU1, and ‐OC43 usually cause only mild‐to‐moderate respiratory diseases with a seasonal pattern, whereas severe acute respiratory syndrome coronavirus (SARS‐CoV), Middle East respiratory syndrome coronavirus (MERS‐CoV) and severe acute respiratory syndrome coronavirus 2 (SARS‐CoV‐2) have sequentially emerged in the past two decades to cause mild‐to‐severe respiratory diseases and have high mortality rates.^[^ ^1^ ^]^ While SARS‐CoV spread only between November 2002 and August 2003 across 32 countries/regions, leading to a total of 8422 probable cases and 919 SARS‐related deaths,^[^ ^2^ ^]^ MERS‐CoV has continued to cause infections since its first identification in 2012, though largely confined within the Eastern Mediterranean region, resulting in a total of 2591 confirmed cases and 894 deaths as of August 2022 (https://www.emro.who.int/health‐topics/mers‐cov/mers‐outbreaks.html). No licensed vaccine is available for MERS‐CoV, despite its unusually high fatality rate of ≈ 35%. SARS‐CoV‐2, the causative agent of coronavirus disease 2019 (COVID‐19), has caused a devastating global pandemic that is still ongoing since its first emergence in late‐December 2019 in Wuhan,^[^ ^3^ ^]^ causing > 770 million confirmed cases and over 6.9 million deaths as of September 2023 (https://covid19.who.int/). Remarkably, SARS‐CoV‐2 has rapidly evolved from its ancestral strain into a large number of variants, several of that have been designated variants of concern (VoCs) (https://www.cdc.gov/coronavirus/2019‐ncov/variants/variant‐classifications.html). These new variants have demonstrated an increasing capacity for antibody evasion and transmission, leading to reductions in the effectiveness of vaccines in current use,^[^ ^4^, ^5^, ^6^, ^7^, ^8^, ^9^, ^10^ ^]^ thus putting numerous lives at risk of severe disease and death. Therefore, there is an urgent need for vaccines that can offer broad protection against MERS‐CoV, SARS‐CoV‐2 variants, and emerging zoonotic coronaviruses.

Traditionally, vaccines are whole‐organism based on attenuated or inactivated pathogens, but they are subject to potential issues of safety, efficacy, manufacturing and cost‐effectiveness.^[^ ^11^ ^]^ Hence, the focus of vaccine development has shifted to subunit vaccines that are based on pathogen‐derived antigenic components. Most approved or authorized COVID‐19 vaccines are subunit vaccines based on the surface spike (S) glycoprotein of SARS‐CoV‐2.^[^ ^12^ ^]^ S contains a receptor‐binding domain (RBD) that initiates viral entry into the host cell by interacting with the host angiotensin converting enzyme 2 (ACE2) receptor.^[^ ^13^ ^]^ Multiple studies have shown that RBD is the major target for potently neutralizing antibodies (nAbs), mainly through directly preventing binding of S to ACE2 on the host cell and hence blocking infection,^[^ ^7^, ^14^, ^15^, ^16^ ^]^ whereas some possess cross‐reactivity by binding to a conserved epitope and may function to destabilize the trimeric S.^[^ ^17^, ^18^ ^]^ Similar to SARS‐CoV‐2, both MERS‐CoV and SARS‐CoV initiate infections of host cells through the interaction of their surface S with their corresponding entry receptors, being dipeptidyl‐peptidase 4 (DPP4)^[^ ^19^ ^]^ and ACE2,^[^ ^20^ ^]^ respectively. Both MERS‐CoV and SARS‐CoV possess a spike RBD that shares an amino acid similarity of ≈17% and 73% to SARS‐CoV‐2 RBD, respectively. Previous studies showed that combining RBD of different species of CoVs could confer the advantage of overcoming immunodominance effects and directing the generation of nAbs toward conserved epitopes and hence allowing them to gain cross‐reactivity.^[^ ^21^, ^22^ ^]^

It has been proposed that the multivalency of the immunogen is important for inducing robust B‐cell responses by triggering cross‐linking of B‐cell receptors.^[^ ^23^ ^]^ This has been supported by an earlier report that showed that I53‐50 nanoparticles multivalently displaying SARS‐CoV‐2 RBD on their surface were able to elicit potent nAb responses whilst monomeric RBD was hardly effective.^[^ ^24^ ^]^ I53‐50 subunits can self‐assemble into a 120‐meric icosahedral nanoparticle,^[^ ^25^ ^]^ whilst mi3^[^ ^26^ ^]^ and ferritin subunits^[^ ^27^ ^]^ can respectively form 60‐meric dodecahedral and 24‐meric octahedral nanoparticles, allowing for multimeric antigen display. Ferritin nanoparticles have been used as an antigen‐presenting vehicle to elicit robust antibody responses against Epstein‐Barr Virus,^[^ ^28^ ^]^ influenza A,^[^ ^29^ ^]^ respiratory syncytial virus^[^ ^30^ ^]^, and SARS‐CoV‐2.^[^ ^21^ ^]^

Although previously studies have demonstrated the effectiveness of mosaic nanoparticles displaying the RBDs from SARS‐CoV‐2 and seven other animal sarbecoviruses,^[^ ^21^, ^31^ ^]^ it remains unclear whether mosaic nanoparticles co‐displaying the RBDs from sarbecoviruses (e.g., SARS‐CoV and SARS‐CoV‐2) and a merbecovirus (MERS‐CoV), which share low degrees of similarity, can promote the elicitation of strong cross‐reactive antibody responses and offer sufficient protection against these coronaviruses. Here, we report the immunogenicity and viral challenge studies to evaluate a mosaic RBD nanoparticle vaccine candidate, with the RBD of SARS‐CoV, SARS‐CoV‐2, and MERS‐CoV being simultaneously displayed on the rationally designed Helicobacter pylori‐bullfrog hybrid ferritin nanoparticle.^[^ ^28^ ^]^ This heterotypic mosaic nanoparticle induced potent and broad‐spectrum antibody responses against SARS‐CoV, SARS‐CoV‐2, and MERS‐CoV in both mice and cynomolgus macaques. It conferred effective protection of the immunized mice from viral challenges of all three types of betacoronavirus. It was able to protect mice from the challenge of SARS‐2 VOCs 5 months after the booster was given. Moreover, sera from these immunized mice were able to cross‐react and cross‐neutralize multiple sarbecoviruses. These results therefore highlight the potential of this RBD mosaic nanoparticle as a promising vaccine candidate and provide a framework for the rational design of next‐generation coronavirus vaccines for combating emergent zoonotic coronaviruses.

## 2 Results

### 2.1 Design, In Vitro Assembly, and Characterization of RBD Nanoparticles

**Figure 1:**
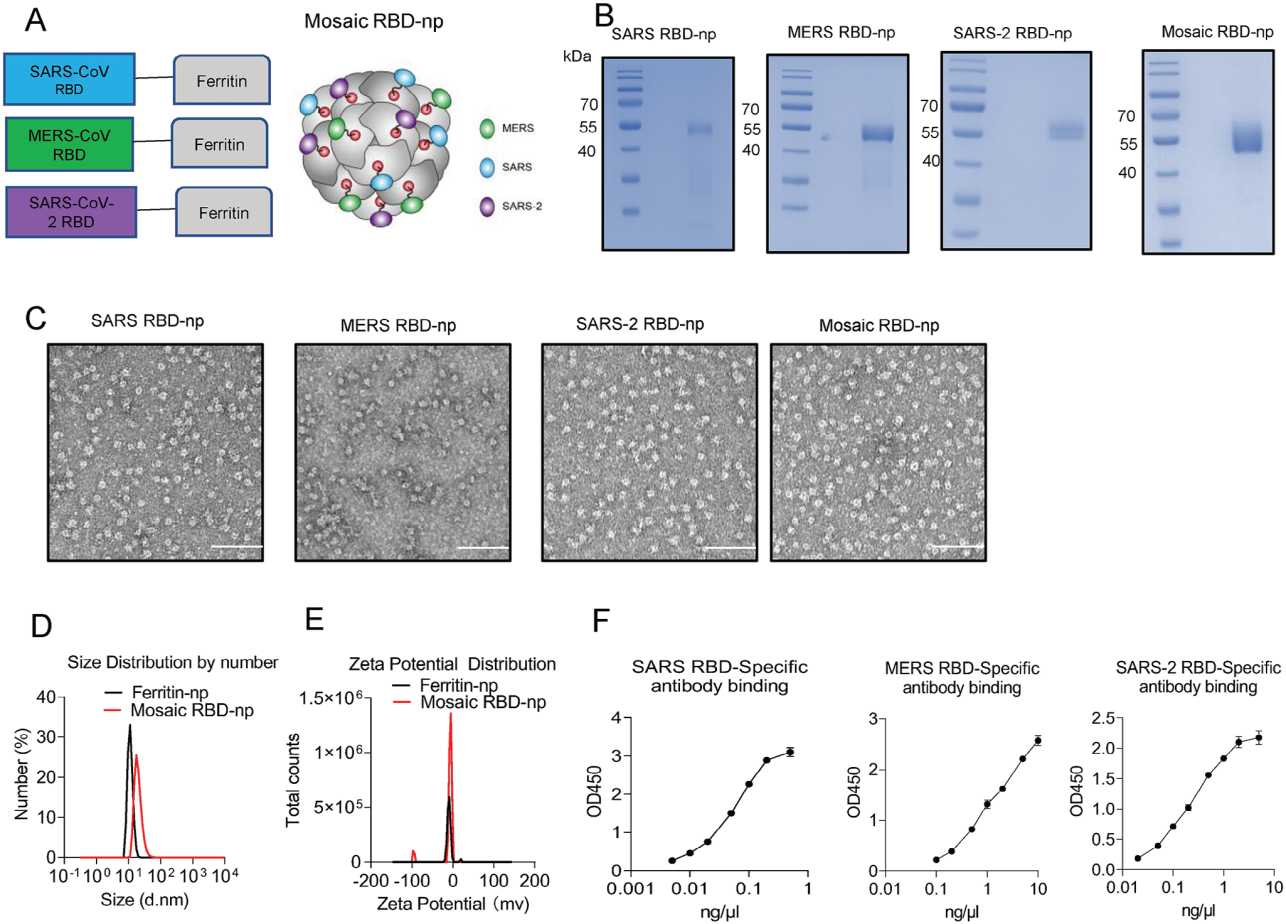
Construction and characterization of mosaic‐RBD nanoparticles. A) Schematic of nanoparticles components: MERS‐CoV, SARS‐CoV, and SARS‐CoV‐2 RBD were fused to the N terminal of the recombinant ferritin. B) SDS‐PAGE/commissa blue staining of nanoparticles. C) Negative‐stain electron micrographs of purified nanoparticle immunogens. Scale bar, 50 nm. D) Size distribution analysis of Mosaic‐RBD nanoparticles and recombinant Ferritin nanoparticles via DLS. E) Zeta‐potential distribution diagram for the Mosaic‐RBD nanoparticles and recombinant Ferritin nanoparticles. F) the binding assay of Mosaic‐RBD nanoparticles with the SARS‐CoV‐2, SARS‐CoV, and MERS‐CoV RBD‐specific monoclonal antibodies by ELISA.

To enhance immunogenicity, the RBDs of SARS‐CoV, MERS‐CoV (EMC2012), and SARS‐CoV‐2 (WT) were multivalently displayed on the exterior surface of the ferritin nanoparticle (np). To do this, each of the RBD was genetically fused to the N‐terminal of Helicobacter pylori‐bullfrog hybrid ferritin nanoparticle through a (SG3)_2_ linker to construct the fusion protein components. The sequences of ferritin nanoparticles are listed in Table S1 (Supporting Information). To obtain homotypic RBD‐np, the corresponding plasmid construct was transfected individually; to obtain mosaic RBD‐np, plasmids encoding all three types of RBD‐np fusion proteins were co‐transfected in an equal molar ratio. The RBD‐np constructs were recombinantly expressed using mammalian (Freestyle 293 F) cells, and the RBD‐np fusion proteins were secreted into the medium for assembly into nanoparticles (**Figure** **1**A). Assembled nanoparticles were purified via affinity chromatography. Coomassie blue staining of the SDS‐PAGE gel revealed a major band of the subunits of the mosaic nanoparticles with a molecular mass ≈ 55 kDa; a broader band resulted from the co‐transfection of three constructs suggests that co‐display of different RBDs (Figure 1B). Transmission electron microscopy (TEM) images showed that both of homotypic and mosaic nanoparticles were able to self‐assemble into cage‐like particles (Figure 1C). Dynamic light scattering (DLS) showed that the average diameter of a mosaic RBD‐np was 17.6 nm, which was greater than that of a naked ferritin nanoparticle (without RBD fusion) that was 11.25 nm (Figure 1D). The zeta‐potential of mosaic RBD‐np (−4.93 ± 3.54 mV) was similar to that of naked ferritin nanoparticle (−8.75 ± 3.82 mV) (Figure 1E), suggesting the display of RBDs did not affect the nanoparticle stability. The DLS analysis in intensity for the mosaic RBD‐np is supplied in Figure S1A (Supporting Information). The co‐display of RBDs derived from MERS‐CoV, SARS‐CoV, and SARS‐CoV‐2 on the mosaic RBD‐np was verified by the binding of monoclonal antibodies specific to each of the three RBDs by ELISA and BLI experiments (Figure 1F, Figure S1B, Supporting Information).

### 2.2 Mosaic RBD Nanoparticle Vaccine Elicited Robust Antibody Responses in BALB/c Mice

**Figure 2:**
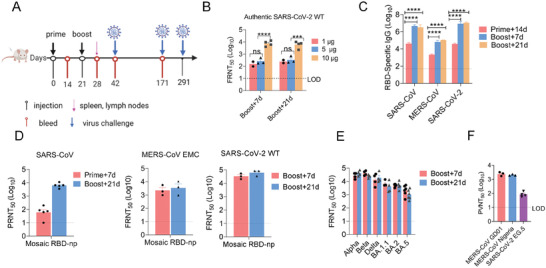
Immunization of Mosaic RBD‐np induce robust humoral response in mice. A) Scheme of the immunization process in mice. B) Six‐week‐old female BALB/c mice were immunized with 1, 5, or 10 µg mosaic RBD‐np. Mice were boosted 3 weeks with their respective antigen dose after primary vaccination. Neutralization antibody were detected by focus reduction neutralization test (FRNT) on day 7 and day 21 after boost immunization. n = 2, 3 or 4, Bars indicate median values. C) Six‐week‐old female BALB/c mice were immunized with 10 µg mosaic RBD‐np/SAS. Mice were boosted 3 weeks with their respective antigen dose after primary vaccination. IgG responses in the sera of vaccinated mice were evaluated two weeks after priming or boosting by ELISA for binding to SARS‐CoV‐2 (WT), MERS‐CoV and SARS‐CoV RBDs. n = 3 mice per group, and the serum were pooled by group. Three independent experiments were analysed. D) Neutralization antibody titre of SARS‐CoV were detected by plaque reduction neutralization test (PRNT). Neutralization antibody titre of SARS‐CoV‐2 WT and MERS‐CoV EMC were detected by FRNT. n = 3‐5 mice per group. E) Neutralization antibody titre of SARS‐CoV‐2 VOCs were detected by FRNT. n = 3‐5 mice per group. F) Neutralization titre of MERS‐CoV GD01, Nigeria, and SARS‐rCoV WIV‐1, Pangolin were detected by pseuovirus assay. Statistical analyses were performed using two‐way ANOVA. Data represent two independent experiments. All results are expressed as mean ± SEM. ns, no significantly difference, ^*^ *p* < 0.05, ^**^ *p* < 0.01, ^***^ *p* < 0.001, ^****^ *p* < 0.0001.

To evaluate the efficacy of mosaic RBD‐np immunization, 6–8‐week‐old female BALB/c mice were immunized twice by intramuscular injection at a 3‐week interval (**Figure** **2**A). To determine the appropriate antigen dose for immunization, mice were immunized with 1, 5, or 10 µg mosaic RBD‐np. We examined the serum neutralization titers against the SARS‐CoV‐2 WT strain by focus reduction neutralization test (FRNT) 7 and 21 days after booster (Figure 2B). At both time points, vaccination with 10 µg mosaic RBD‐np induced significantly higher antibody titers than dosing with 1 or 5 µg, so 10 µg was chosen as the dose throughout the rest of the experiments in the presence of Sigma adjuvant system (SAS). Control animals were sham‐immunized with PBS also in the presence of SAS, their induced responses were below the limit of detection (LOD); thus, throughout the rest of the text, the level of LOD indicates the level of sham control.

The RBD‐specific IgG binding activities of the immune serum were examined by ELISA (Figure 2C). Strong antibody responses against all three types of RBD were detected 14 days after priming, and significantly higher levels were induced after a booster was given. Neutralization titers of the immune serum were measured by plaque reduction neutralization test (PRNT) for SARS‐CoV, and by FRNT for MERS‐CoV and SARS‐CoV‐2. Very high titers elicited against all three viruses were detected 21 days after the booster (Figure 2D; Table S2A, Supporting Information). The immune serum was potently cross‐neutralizing to a variety of VOCs, including Alpha, Beta, Delta, BA.1.1, BA.2, and BA.5 (Figure 2E; Figure S2A, Supporting Information), and other MERS‐CoV strains ChinaGD01 and Nigeria (Figure 2F; Table S2B, Supporting Information). Titers against BA.1.1, BA.2, and BA.5 Omicron variants were lower than against WT, consistent with other studies.^[^ ^8^, ^32^, ^33^ ^]^ Moreover, we found that the vaccine serum retained neutralizing activity against EG.5, the currently circulating SARS‐CoV‐2 variant (Figure 2F).

### 2.3 Mosaic RBD Nanoparticle Vaccine Induced Th1‐Biased Immune Response in Mice

**Figure 3:**
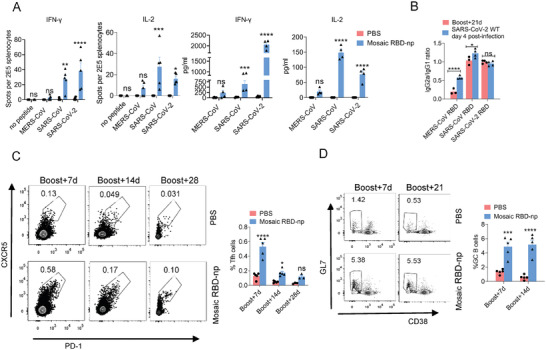
Immunization of Mosaic RBD‐np induce Th‐1 biased cellular and humoral response in mice. A) Mice were immunized with 10 µg mosaic RBD‐np/SAS, the control group were immunized with PBS/SAS. Spleens were collected at 14 days post‐boost immunization. ELISPOT was used to determine the abundance of IFN‐γ^+^, IL‐2^+^ splenocytes (2 × 10^5^ splenocytes/well) after 24 h stimulation with peptide pool. ELISA was used to determine the level of IFN‐γ, IL‐2 in the cell supernatant after 24 h stimulation with peptide pool n = 5 mice per group. Statistical analyses were performed using two‐way ANOVA. B) Ratio of MERS‐CoV, SARS‐CoV, and SARS‐CoV‐2 RBDs binding IgG2a to IgG1 were determined by ELISA at day 21 after boost immunization and at day 4 after SARS‐CoV‐2 WT infected. n = 3 mice per group, and the serum were pooled by group. Three independent experiments were analysed. Statistical analyses were performed using two‐way ANOVA. C‐D) Draining lymph nodes were collected at day 7, day 14, day 21 and day 28 after the boost immunization. The percentages of Tfh cells C) and GC cells D) were determined by FCS. n = 3‐5 mice per group. Statistical analyses were performed using two‐way ANOVA. All results are expressed as mean ± SEM. ns, no significantly difference, ^*^ *p* < 0.05, ^****^ *p* < 0.0001.

To assess cellular responses induced by the RBD‐np vaccine, splenocytes collected at day 14 post‐booster were stimulated with three peptide libraries spanning the full length of the RBD of MERS‐CoV, SARS‐CoV, and SARS‐CoV‐2, respectively. IFN‐γ and IL‐2 expression levels were evaluated by both ELISpot and ELISA (**Figure** **3**A). Both assays showed that substantial levels of IFN‐γ and IL‐2 were produced against the SARS‐CoV and SARS‐CoV‐2 peptide pools whilst minimal responses against the MERS‐CoV pool, suggesting that MERS‐CoV RBD contains no dominant T cell epitopes in BALB/c mice.

To evaluate the Th1/Th2 polarization induced by the mosaic RBD‐np vaccine, we examined the IgG2a/IgG1 ratio of RBD‐binding antibodies by ELISA. Since IgG1 production is non‐specific whilst IgG2a production is a marker for Th1 response, a higher IgG2a/IgG1 ratio is indicative of a Th1‐skewing immune response. Interestingly, while there was no biased response observed for MERS‐CoV RBD at 21 days after boosting, significantly higher IgG2a/IgG1 ratios were detected post‐challenge with SARS‐CoV‐2 WT strain (Figure 3B). By contrast, comparably high IgG2a/IgG1 ratios were maintained for SARS‐CoV and SARS‐CoV‐2 before and after the viral challenge.

As CD4^+^ T follicular helper (Tfh) cells are essential for the formation and maintenance of germinal centers (GCs) where affinity maturation, selection, and differentiation of B cells occur, we monitored the levels of Tfh cells after boosting (Figure 3C; Figure S3, Supporting Information). Significantly higher proportions of Tfh cells were observed 7 days post mosaic RBD‐np vaccine booster compared to sham control. In line with the elevated levels of Tfh cells, there was a substantial expansion of GC B cell population within the lymph nodes 7 days after boosting. While at 14 days after boosting the percentage of Tfh cells markedly reduced by 3.5‐fold, and a similar level of GC B cells was observed (Figure 3C,D; Figure S3, Supporting Information).

### 2.4 Mosaic RBD Nanoparticle Immunization Induced Protective Immunity Against Viral Challenge of SARS‐CoV, MERS‐CoV, and SARS‐CoV‐2 Variants in Mice

**Figure 4:**
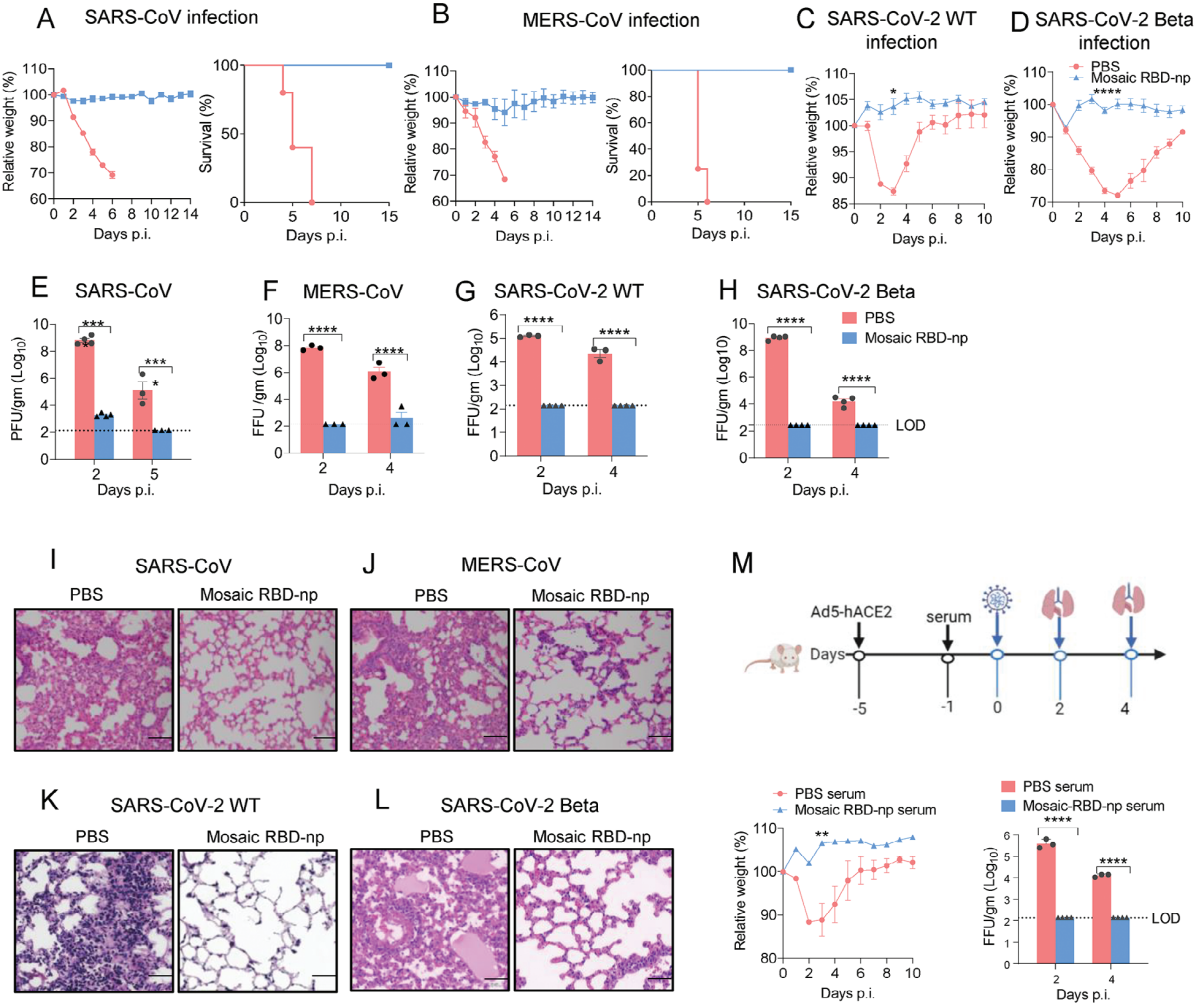
Mosaic RBD‐np immunization provides protection against the SARS‐CoV, MERS‐CoV, SARS‐CoV‐2 WT, and SARS‐CoV‐2 Beta challenge in mice. Challenges were conducted on day 21 post‐final immunization. A) Mice were intranasally infected with 500 PFU SARS‐CoV mouse‐adapted virus. Mortality and weight were monitored daily until day 14 post‐infection B) hDPP4–KI mice were infected with 1000 FFU MERS‐CoV mouse‐adapted virus. Mortality and weight were monitored daily until day 14 post‐infection. n = 5 mice per group. C) Five days after transduction with 2.5 × 10^8^ PFU of Ad5‐hACE2, mice were intranasally infected with 1 × 10^5^ FFU of SARS‐CoV‐2 WT; Immunized mice were directly infected with 1 × 10^5^ PFU of SARS‐CoV‐2 Beta D). Weight was monitored daily until day 10 post‐infection. E–H) To obtain virus titres, lungs were homogenized at the indicated time points and tittered on Vero E6 cells. Titres are expressed as PFU/g or FFU/g tissue. (n = 3–6 mice per group per time point). Statistical analyses were performed using two‐way ANOVA. I–L) Sections of paraffin embedded lungs from infected mice were stained with hematoxylin/eosin at day 4 or 5 post‐infection. Scale bar = 50 um. (m) Four days after transduction with 2.5 × 10^8^ PFU of Ad5‐hACE2, mice were intravenous injected with 150 µl serum from mice immunized with PBS, mosaic RBD‐np/SAS. 24 h later, mice were infected with 1 × 10^5^ FFU of SARS‐CoV‐2 WT. Weight was monitored daily until day 10 post‐infection. Viral load in lung is expressed as FFU/g tissue. (n = 3–6 mice per group per time point). Statistical analyses were performed using two‐way ANOVA.

To evaluate the in vivo efficacy of mosaic RBD‐np, we evaluated the protections from the viral challenge of SARS‐CoV, MERS‐CoV, and SARS‐CoV‐2 variants in various mouse models. BALB/c mice were challenged with mouse‐adapted SARS‐CoV, while KI‐hDPP4 mice were challenged with mouse‐adapted MERS‐CoV. Ad5‐human ACE2 (hACE2)‐transduced mice, as generated by transduction of BALB/c mice with Ad5‐hACE2 at day 21 after boost immunization, were challenged with SARS‐CoV‐2 WT strain five days later when hACE2 was sufficiently expressed.^[^ ^34^ ^]^ BALB/c mice, rather than hACE2 transduced mice, were challenged with SARS‐CoV‐2 Beta strain, as this strain contains a N501Y mutation in spike RBD that allows infection via mouse ACE2.^[^ ^35^ ^]^ Mice in each cohort were evaluated for survival, weight loss (**Figure** **4**A–D), viral loads in lung tissues as quantified by plaque‐forming assays or focus‐forming assays (Figure 4E–H), and histopathology (Figure 4I–L). Sham‐immunized mice infected with SARS‐CoV and MERS‐CoV suffered from substantial weight loss and died within 5–6 days post‐challenge. Control animals infected with SARS‐CoV‐2 WT and Beta strains experienced substantial weight loss, approaching 15% and 30% respectively, within the first 3–5 days upon challenge but then underwent self‐recovery. By contrast, vaccination with mosaic RBD‐np provided robust protection against viral challenge so that no weight loss or death was observed. Examination of the post‐mortem lungs of infected mice found that high viral loads were detected in all control groups, whilst no detectable live virus was detected in the lungs of immunized mice except that a small amount of virus was detected 2 days after mice were challenged with SARS‐CoV but the virus was undetectable 3 days thereafter. Extensive lung lesions were observed in the PBS‐treated mice challenged with all three types of viruses; substantial lymphoid infiltration was found in mice infected with SARS‐CoV‐2 WT strain, and peribronchial lymphoid infiltration with progression to an interstitial pneumonia was found in case of Beta infection. By contrast, there was little pathological change in immunized mice infected with SARS‐CoV and SARS‐CoV‐2 WT strain; some lymphoid infiltration was found in those infected with MERS‐CoV, and only mild lung injury with moderate lymphoid infiltration was detected in the Beta infection group.

### 2.5 Mosaic RBD‐np Vaccine‐Induced Sera Limited SARS‐CoV‐2 Infection

To investigate the contribution of the humoral response to vaccine‐mediated protection, we intravenously transferred serum collected from immunized and sham‐immunized mice into hACE2‐transduced mice 1 day before the challenge with SARS‐CoV‐2 WT strain (Figure 4M). In contrast to the control group in which an up to 10% weight loss was observed in the first three days upon challenge, viral infection was limited by pre‐treatment with anti‐sera, and no weight loss was resulted. In addition, no live virus was detected in the lungs of the mice pre‐treated with anti‐serum, whilst high viral loads were found in the control group.

### 2.6 Mosaic RBD Nanoparticle Vaccine Induces Long‐Lived Antibody Responses and Protection

**Figure 5:**
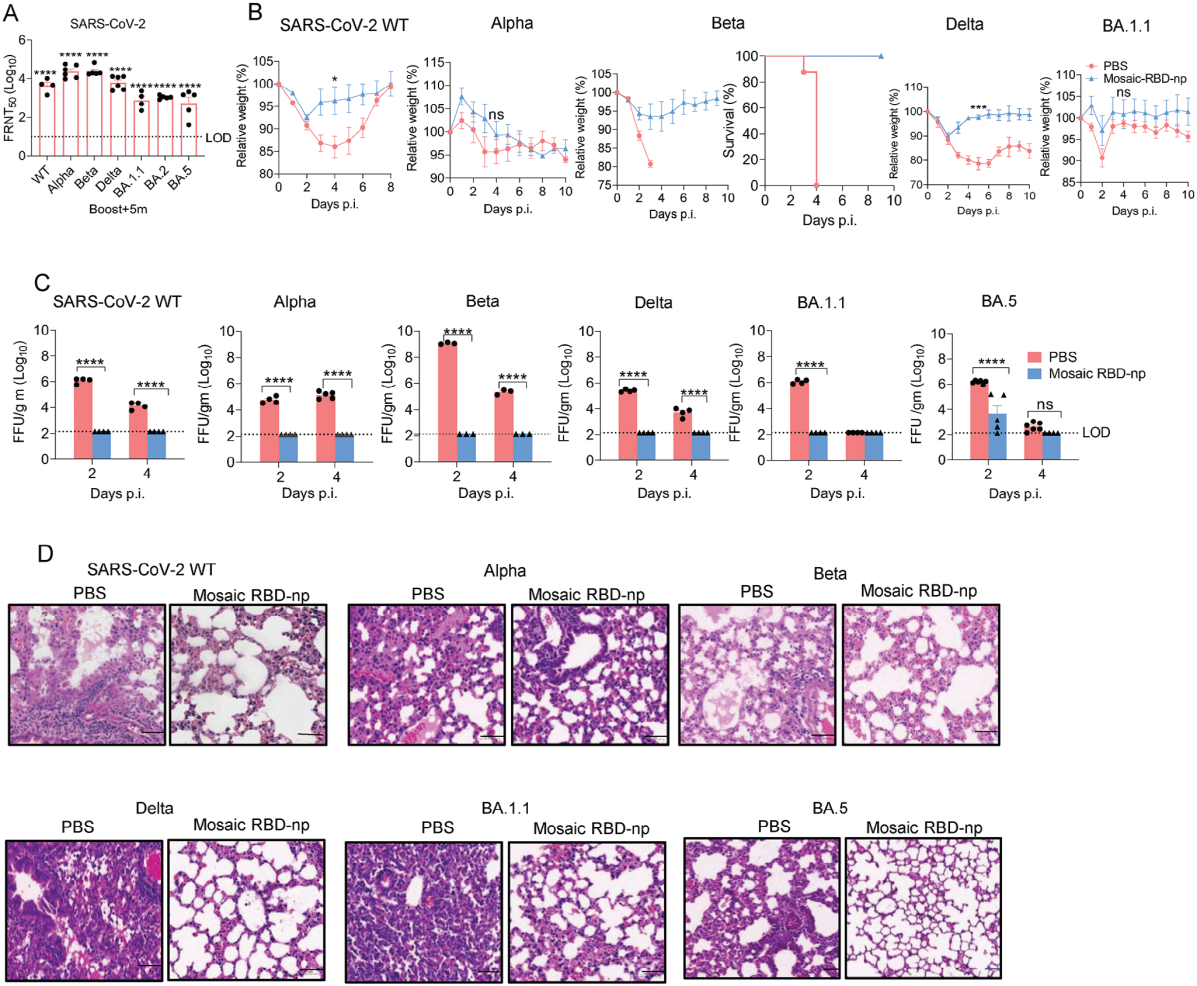
Durable immune response and protection induced by mosaic RBD‐np/SAS immunization from SARS‐CoV‐2 WT, and VOCs challenge. Five months (9 months for BA.5) after the final immunization, mice were infected with SARS‐CoV‐2 WT and VOCs (Alpha, Beta, Delta, BA.1.1, and BA.5), and serum were collected before the infection to detect the neutralization titre by FRNT. A) Neutralization titres of SARS‐CoV‐2 after 5 months of boost immunization were detected by FRNT. n = 3 mice in PBS group; n = 4‐6 mice in mosaic RBD‐NP/SAS group. B) Five months after boost immunization, mice were transduced with 2.5 × 10^8^ PFU of Ad5‐hACE2. Five days later, mice were intranasally infected with B) 1 × 10^5^ FFU of SARS‐CoV‐2 WT, 5 × 10^4^ FFU of SARS‐CoV‐2 Delta; Mice were directly infected with 5 × 10^4^ FFU of SARS‐CoV‐2 Alpha, Beta, BA.1.1, and BA.5. Weight was monitored daily until day 10 post‐infection. C) Viral load in lung is expressed as FFU/g tissue. n = 3‐4 mice per group. D) Sections of paraffin embedded lungs from infected mice were stained with hematoxylin/eosin at day 4 post‐infection. Scale bar = 50 um. All results are expressed as mean ± SEM. Statistical analyses were performed using two‐way ANOVA.

To investigate the durability of antibody responses and protection elicited by the mosaic RBD‐np, serum samples collected from immunized mice 5 months (9 months for BA.5) after booster were used to test neutralization activities against SARS‐CoV‐2 variants. There was no significant reduction in neutralization titres against Alpha, Beta, Delta, and BA.5 compared to 21 days post‐booster. Interestingly, significant waning was observed for WT, BA.1.1, and BA.2 although titres against these strains remained substantially higher than sham control (**Figure** **5**A; Figure S4, Supporting Information). Challenge experiments showed that there were limited weight changes in all immunized mice, even though the control mice developed severe disease upon certain infections (Figure 5B): significant weight loss of up to 15% resulted after WT strain infection, and recovery was prolonged, and rather than causing only substantial weight loss, the Beta strain became lethal (Figure 5B). Lung viral loads were below the level of detection in all immunized mice whilst sham‐immunized mice showed high titres (Figure 5C). In line with previous reports that Omicron is less pathogenic in mice,^[^ ^36^ ^]^ lung viral loads in the BA.1.1 and BA.5 infection groups were below or close to the limit of detection 4 days post‐challenge. Extensive lesions with lymphoid infiltration were observed in the lungs of all control mice and signs of interstitial pneumonia and edema seen in the lungs of mice infected with WT, Alpha, Beta, and Delta strains. By contrast, only mild lymphoid infiltration was found in the lungs of immunized mice (Figure 5D).

### 2.7 Mosaic RBD Nanoparticle Vaccine Induces Robust Antibody Responses in Non‐Human Primates

**Figure 6:**
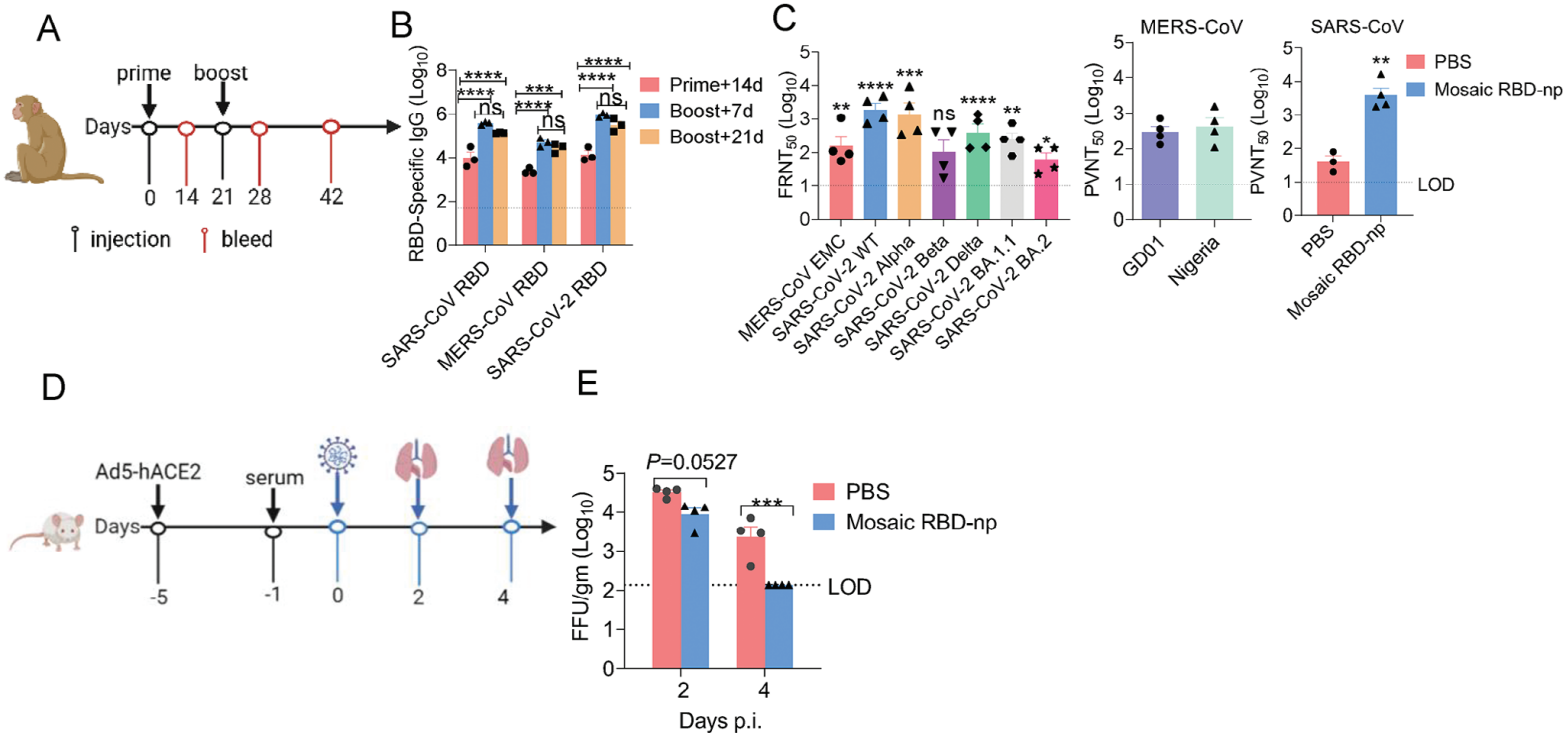
Robust antibody response induced by mosaic in cynomolgus monkeys. A) Immunization and sample collection procedures for cynomolgus monkeys. B) SARS‐CoV, MERS‐CoV, and SARS‐CoV‐2 RBDs‐specific IgG titres at the indicated time points. Data shown are geometric means ± SEM. n = 3 monkeys in PBS/SAS group; n = 4 monkeys in mosaic RBD‐np/SAS group. Plasma was pooled by group. Three independent experiments were analyzed. Statistical analyses were performed using two‐way ANOVA. C) Neutralization titres against SARS‐CoV‐2 WT, SARS‐CoV‐2 VOCs, and MERS‐CoV EMC at the indicated time points using FRNT. Neutralization titres against MERS‐CoV GD01 and Nigeria, SARS‐CoV at the indicated time points using the pseudovirus assay. D) Four days after transduction with 2.5 × 10^8^ PFU of Ad5‐hACE2, mouses were intravenous injected with 150 µl plasma from monkeys immunized with PBS/SAS, mosaic RBD‐np/SAS. 24 h later, mice were infected with 1 × 10^5^ FFU of SARS‐CoV‐2 WT. E) Viral load in lung is expressed as FFU/g tissue. Statistical analyses were performed using two‐way ANOVA.

To further evaluate the efficacy of the mosaic RBD‐np vaccine, we conducted immunization studies in non‐human primates (NHPs). Four cynomolgus macaques (female, 12–16 years old) were immunized with the mosaic RBD‐np adjuvanted with SAS, followed by a booster on day 21 (**Figure** **6**A). Temperature was monitored and measured daily for 4 days ( Figure S5A, Supporting Information). Little alteration in body temperature was observed, suggesting mosaic RBD‐np had not triggered side effects that resulted in fever or hypothermia. Vaccination elicited high titres of IgG antibodies against SARS‐CoV, MERS‐CoV, and SARS‐CoV‐2 RBDs 14 days post‐prime with significantly higher levels found at 7 days post‐booster (Figure 6B). Similarly high levels were detected at 21 days post booster. MERS‐CoV, SARS‐CoV, and SARS‐CoV‐2 variants were used to evaluate the neutralization activity of immune sera. Significantly higher titres were elicited compared to the control group, including Omicron BA.2 (Figure 6C). Although higher titres were observed for the SARS‐CoV‐2 Beta strain, the difference from the control group was not significant since the two serum samples had poor neutralization activities against the Beta strain. Using pseudoviral neutralization assays we showed that the NHP immune serum was also able to effectively neutralize MERS‐CoV GD01 and Nigeria strains, as well as SARS‐CoV (Figure 6C, Figure S5B, Supporting Information). NHP anti‐serum intravenously transferred to hACE2‐transduced mice 1 day before the viral challenge of 1 x 10^5^ FFU SARS‐CoV‐2 WT strain was able to clear live virus from lungs within 4 days post‐infection (Figure 6D,E).

### 2.8 Mosaic RBD Nanoparticle Vaccine Induces Cross‐Reactive B Cell Responses in Mice

**Figure 7:**
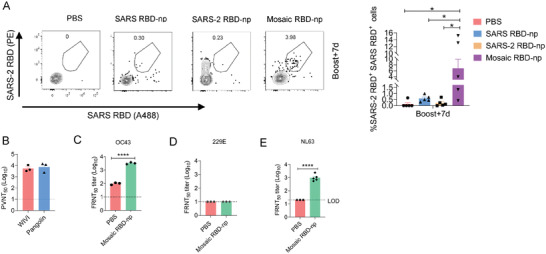
Mosaic RBD‐np immunization induces cross‐ reactive RBD‐specific B cell response between SARS‐CoV and SARS‐CoV‐2 and the broad‐spectrum antibody response. A) Draining lymph nodes were collected at day 7 after the boost immunization. RBD‐specific B cell analysis from animals immunized with heterotypic mosaic‐RBD nanoparticles or homotypic RBD nanoparticles (SARS‐CoV RBD‐np and SARS‐CoV‐2 RBD‐np). GC B cells stained with SARS‐CoV RBD‐A488 and SARS‐CoV‐2 RBD‐PE. n = 4‐5 mice per group. All results are expressed as mean ± SEM. Statistical analyses were performed using two‐way ANOVA. B) Neutralization titers of immunized serum against SARS‐related coronavirus WIV‐1 and pangolin were detected by pseudovirus assay. C–E) Neutralization titers of immunized serum against OC43, 229E and NL63 were detected by FFA, IFA.

Mosaic RBD nanoparticle induced cross‐reactive neutralization antibodies in mice and cynomolgus macaques (Figures 2 and 6). Therefore, we next assessed whether there were qualitative differences in B cell responses by assessing the cross‐reactivity of RBD‐specific B cells in mice immunized with homotypic and heterotypic RBD‐nanoparticles. Lymph node cells were collected after two immunizations with SARS‐CoV RBD–np or SARS‐CoV‐2 RBD‐np alone or mosaic RBD–np and were probed with two distinct RBDs (SARS‐CoV and SARS‐CoV‐2) to identify RBD‐specific B cells by flow cytometry. Consistent with previous studies, B cells stained with both SARS‐CoV‐2 RBDs and SARS‐CoV RBDs were detected in the majority of mice immunized with mosaic nanoparticles (**Figure** **7**A; Figure S6, Supporting Information). Next, we want to know whether this cross‐reactive B cell response could induce neutralization antibodies against SARS‐like coronaviruses, such as pangolin and WIV‐1 whose RBD share % and 94.6% amino acid identity to that of SARS‐CoV, respectively. From the pseudovirus neutralization assay, the serum of mice immunized with mosaic RBD‐np had exhibited high neutralization level against the pangolin and WIV‐1 strains (Figure 7B). Interestingly, we also found that sera from mice immunized with mosaic RBD‐np could neutralize OC43, NL63, but not 229E (Figure 7C–E), indicating mosaic RBD nanoparticles immunization could induce broad‐spectrum neutralizing antibodies against various coronaviruses.

## 3 Discussion

Enormous efforts have been put into the development of effective vaccines for combating the global COVID‐19 pandemic. About a year after the outbreak of an epidemic, mRNA‐based vaccines BNT162b2 (Pfizer‐BioNTech) and Moderna (mRNA‐1273) received emergency use authorization from the Food and Drug Administration (FDA) and have been shown to effectively reduce hospitalization and death.^[^ ^37^ ^]^ In contrast to the traditional inactivated or live‐attenuated vaccines that involve the use of the whole pathogens, these mRNA vaccines and many other new ones currently under development and clinical trials^[^ ^38^ ^]^ belong to protein subunit vaccines that contain only the antigenic components of the pathogen.^[^ ^39^ ^]^ Although spike has been widely used as the immunogen for COVID‐19 vaccines due to its high immunogenicity, the receptor‐binding domain of spike is the main target for the most potently neutralizing antibodies and therefore has also garnered much attention.^[^ ^40^ ^]^

As SARS‐CoV‐2 continues to evolve, especially after the identification of Omicron as a VOC, a succession of new variants is emerging at an unprecedented pace with increasing immunoevasive capacities (https://www.biorxiv.org/content/10.1101/2022.09.15.507787v4). In addition to SARS‐CoV‐2, MERS‐CoV, and SARS‐CoV are also highly pathogenic coronaviruses capable of causing severe disease and death, but as yet there is no licensed vaccine available for either of them. Moreover, zoonotic spill‐over may occur so that new coronaviruses may be transmitted to humans, either directly from a natural reservoir such as bats or through an intermediate host, and cause future devastating pandemics like COVID‐19. Therefore, there is an urgent need for the development of broad‐spectrum vaccines that are capable of conferring protection against other coronaviruses. Multivalent antigen presentation on self‐assembling protein scaffolds such as ferritin may be explored as a powerful platform that enables simultaneous display of multiple immunogens allowing for robust immune responses in addition to induction of cross‐reactivity.

Here, we report the immunogenicity and virus challenge studies of a mosaic ferritin nanoparticle vaccine candidate that co‐displays the RBD of SARS‐CoV, MERS‐CoV, and SARS‐CoV‐2. Our results indicate that immunization of this mosaic RBD‐np vaccine is able to trigger a Th1‐skewed immune response and elicit potent neutralizing antibody responses against not only the viral species where the immunogens derived but also a number of other MERS‐CoV strains, SARS‐CoV‐2 variants, and SARS‐related CoVs. Immunized mice are efficiently protected from the viral challenge of SARS‐CoV, MERS‐CoV, and SARS‐CoV‐2 variants, so that lung viral loads are reduced to undetectable levels and limited pathological changes in lungs are resulted in immunized mice compared to control animals. Transfer of immune serum is also able to render protection from viral infection. Importantly, long‐lived immunity is induced and vaccine efficacy persists for at least 5 months in mice, protecting these immunized mice from severe disease and death as found in control groups. Antibody responses with potent and broad neutralizing activities are elicited by immunization of mosaic RBD‐np in NHP models.

While this manuscript was in preparation, Lee et al.^[^ ^41^ ^]^ reported a mosaic RBD nanoparticle using proliferating cell nuclear antigen (PCNA) subunits as the scaffold to display RBDs derived from α‐ (HKU1 and 229E) and β‐CoV (SARS‐CoV, SARS‐CoV‐2 WT, and Delta strain, MERS‐CoV). Immunization with this immunogen‐induced intergenus cross‐reactive antibodies and protection against SARS‐CoV‐2 challenge. However, in that study neutralization titres against Omicron BA.1 were several orders lower than the wild type, the response against MERS‐CoV was weak and no evidence was shown that this vaccine candidate is able to confer protection against other species including MERS‐CoV. Here, by contrast, neutralization titres against BA.1.1 and BA.2 (induced by RBD‐ferritin‐np) showed < 2‐log reductions compared to the wild‐type strain ( Figure S1, Supporting Information). Since BA.1.1 and BA.2 are immune evasive than Omicron BA.1 ,^[^ ^16^ ^]^ it might imply that RBD‐ferritin‐np is able to elicit stronger cross‐reactive antibody responses than RBD‐PCNA‐np. In addition, we find that the anti‐MERS‐CoV response induced by the RBD‐ferritin‐np is as robust as the response against SARS‐CoV and SARS‐CoV‐2. Further, this strong antibody response induced by the RBD‐ferritin‐np also translates into the 100% protection of mice from MERS‐CoV infections. Taken together, ferritin might be superior to PCNA as a vaccine display scaffold in regard to the strength and cross‐reactivity of the humoral response induced.

### 3.1 Limitations of the Study

In the present study, aged rather than young cynomolgus monkeys were used to evaluate the immunogenicity of mosaic RBD‐np vaccine because of the limited availability of monkeys, but the induced immune responses might be different in young animals. Induction of long‐lived immunity and protection by the mosaic RBD‐np vaccine was only accessed against SARS‐CoV‐2 variants in this study, the protective efficacies against SARS‐CoV and MERS‐CoV require further investigation.

## 4 Experimental Section

### Cell Culture

Vero E6 cells, 293 T cells, Huh7 cells were maintained in high glucose Dulbecco's Modified Eagle Medium (DMEM, Gibco) supplemented with L‐glutamine, sodium pyruvate, and 10% fetal bovine serum (FBS). These cells were cultured at 37 °C and 5% CO_2_ in a humidified atmosphere. Freestyle 293F cell lines were maintained in Freestyle 293 expression medium, and cultured at 37 °C and 8% CO_2_ with 120 rpm in a humidified atmosphere.

### Virus

The SARS‐CoV‐2 variants, including WT, alpha (B.1.1.7), beta (B.1.351), eta (B.1.525), and Omicron (BA.1 and BA.2) were isolated from COVID‐19 patients and preserved in Guangzhou Customs District Technology Center BSL‐3 Laboratory. The SARS‐CoV‐2 delta (B.1.617.2) and Omicron BA.5 strains were presented by the Guangdong Provincial Center for Disease Control and Prevention, China. Experiments related to authentic SARS‐CoV‐2 were conducted in Guangzhou Customs District Technology Center BSL‐3 Laboratory. The MERS‐CoV EMC was isolated from MERS patients and preserved in Guangzhou Customs District Technology Center BSL‐3 Laboratory. Mouse‐adapted SARS‐CoV (MA15) was a kind gift from Dr. Kanta Subbarao (National Institutes of Health, Bethesda, MD). Experiments related to authentic SARS‐CoV were conducted in the University of Iowa BSL‐3 Laboratory.

### Peptide Library

A set of 20‐mer peptides encompassing SARS‐CoV‐2, MERS‐CoV, SARS‐CoV S1 proteins were synthesized and used to stimulate the immune cells of mice.

### Expression Constructs

All genes used in this study were synthesized by GenScript and codon optimized for human hosts. To avoid autoimmune reactions, the sequence of ferritin used in our study was constructed as previously reported. Briefly. To obtain the high‐expression level of MERS‐CoV RBD protein, the sequences of MERS‐CoV RBD used in the study correspond to residues 377–588aa. The SARS‐CoV‐2 RBD sequences used in the study correspond to residues 309–530 and are derived from WT strain. The SARS‐CoV RBD sequences in this study correspond to 305–524. To construct the RBD‐np, the RBD sequence was fused to the 3′ end of the recombinant ferritin with a (SG3)_2_ linker. To obtain the secreted protein, a modified bovine prolactin signal sequence was attached upstream of the RBD.

### Biosynthesis of Recombinant Proteins and Purification

The expression vectors were transiently transfected into Freestyle 293 F cell lines using the PEI transfection reagents. 7 days after transfection, the supernatants were collected and centrifuged to remove the cell ribs. The RBD‐np were purified by the affinity chromatography using strep‐tectin at pH 8.0 (IBA). Purified proteins were resuspended in assembly buffer (20 × 10^−3^ m m Tris–HCl, 50 × 10^−3^ mm NaCl (pH 8.0).

### Characterization of the Mosaic‐RBD‐np

The purified mosaic‐RBD‐np was verified by SDS‐PAGE/Commissa staining. The size of the Mosaic‐RBD‐np and Zeta potential of the cages in assembly buffer were measured by Zetasizer Nano (Malvern Instruments Ltd.) 0.5 mg ml^−1^ samples were absorbed to freshly glow‐discharged carbon‐coated grids, then were rinsed with 2% phosphotungstic acid (PTA) for 5 min. The grid was examined by 100 kv transmission electron microscopy after dried by air.

### Vaccine Formulation

According to the instruction of SAS adjuvant (Sigma). SAS adjuvant was resuspended with 1 ml saline, and 40 °C for 5 min. Then vortex to make SAS adjuvant uniform. Mosaic‐RBD‐np protein mixed with SAS adjuvant in 1:1 volume.

### Mice Immunization and Infection

SARS‐CoV‐2 challenge experiments: 6–8 weeks‐old female BALB/C mice were immunized with 10 µg RBD‐np with SAS adjuvant by intramuscular route. Three weeks later, boost immunization with the same dose. 5 days before the SARS‐CoV‐2 WT/Delta strain infection, mice were lightly anesthetized with isoflurane and transduced intranasally with 2.5 × 10^8^ FFU of Ad5‐hACE2. Five days after transduction, mice were infected 1 × 10^5^ FFU SARS‐CoV‐2 WT or 5 × 10^4^ FFU SARS‐CoV‐2 Delta strain. For SARS‐2 Alpha, Beta, and Omicron BA.1.1 challenge experiments, BALB/C mice were directly infected with 5 × 10^4^ FFU virus in a total volume of 75 µl per mouse via intranasal administration.

MERS‐CoV challenge experiment: KI‐hDPP4 mice were lightly anesthetized with isoflurane and were infected intranasally with mouse‐adapted MERS‐CoV (1000 FFU), in a total volume of 50 µL of DMEM.

SARS‐CoV challenge experiment: 6–8 weeks‐old female BALB/C mice were lightly anesthetized with isoflurane and were infected intranasally with mouse‐adapted SARS‐CoV (MA15500 PFU) in a total volume of 50 µL of DMEM.

### Cynomolgus Monkey Immunization

12–16‐years‐old female cynomolgus monkeys were immunized with 100 µg Mosaic RBD‐np with SAS adjuvant by intramuscular route. Three weeks later, boost immunization with the same dose. After the prime and booster vaccination of the monkey, body temperature was measured every day for a total of 96 h at the anus.

### Detection of Viral Load in Lung

Removed the lung tissue after mice were sacrificed. Lung tissue was homogenized in 1 mL of DPBS medium, and then frozen at −80 °C. After freezing and thawing, lung homogenate was clarified by centrifugation at 5000 g for 10 min at 4 °C. Viral titres in supernatant were determined by Vero E6 cell lines. Briefly, 1.8 × 10^4^ /well cells were seeded on the 96‐well plates. Then, 50 µl of tenfold serially diluted suspension was added to each well, and then incubated at 37 °C for 1 h. Discard the supernatant, and add 100 µl 1.6% CMC in each well. The plates were incubated in a CO_2_ incubator at 37 °C for 24 h, and then the plates were fixed with 4% paraformaldehyde for 30 min. Then the plates were determined as the FFA according to the previous described.

### Focus Formation Assay (FFA)

1.8 × 10^4^ per well Vero E6 cells were seed into 96‐well plate. 16 h later, virus serum mixture or lung homogenate was serially diluted and incubated at 37 °C for 24 h with 5% CO2. 24 h later, plates were fixed with 4% paraformaldehyde for 30 min and permeabilized with 0.2% Triton X‐100. Cells were then stained with rabbit anti‐SARS‐CoV‐2 N protein polyclonal antibody at 37 °C for 24 h. Plates were washed with PBST three times, and followed by an HRP‐labeled goat anti‐rabbit secondary antibody (Cat. No.: 109‐035‐088, Jackson ImmunoResearch Laboratories, Inc. West Grove, PA). The foci were visualized by TrueBlue Peroxidase Substrate (KPL, Gaithersburg, MD), and counted with an ELISPOT reader (Cellular Technology Ltd. Cleveland, OH).

### Histopathology Analysis

On the fourth day after infection, lung tissues were removed and fixed in zinc formalin, and paraffin‐embedded. Sections were stained with hematoxylin/eosin for histological analysis.

### ELISPOT

To detect the specific cellular immune response, IFN‐γ and IL‐2 based ELISPOT assays were performed. According to the manual instructions, 96‐well PVDF membrane‐bottomed plates were used in these experiments. Add 50 µl of diluted coating antibody solution into each well of the ELISPOT plate. Cover the plate with a lid and incubate overnight at 4 °C. Add 2 × 10^5^ mouse splenocytes cells into each well of the plate, and then incubate at 37 °C for 24 h with 5% CO2 and 100% humidity. Then, three peptide pools (MERS‐CoV S1, SARS‐CoV S1, and SARS‐CoV‐2 S1) were apart added to the wells. PMA/ Ionomycin was added as a positive control. Cells incubated without peptide stimulation were employed as a negative control. Remove cells and wash the plates three times with the 0.1% PBST. Add 100 µl of diluted biotinylated detection antibody into each well. Seal the plate with an adhesive cover slip and incubate 2 h at RT. Wash the plates with 1% PBST three times, and then add 100 µl freshly prepared AEC solution into each well. Cover the plate with a lid and incubate for 30 min at RT in the dark. Stop the reaction by emptying the plate and thoroughly rinse both sides of the PVDF membrane with demineralized water. The numbers of the spots were determined using an automatic ELISPOT reader.

### Live SARS‐CoV‐2, MERS‐CoV, and SARS‐CoV Neutralization Assay

The live virus neutralization assay was conducted in a BSL‐3 facility. Briefly, serum from immunized mice was inactivated at 56 °C for 30 min to eliminate the effect of complement. Then the serum was fourfold serially diluted and mixed with the same volume of SARS‐CoV‐2, MERS‐CoV, and SARS‐CoV (200 FFU/well), incubated at 37 °C for 1 h. Then the mixture was abandoned and add 100 µl 1.6% CMC each well. The plates were incubated in a CO2 incubator at 37 °C for 24 h. 24 h later, the plates were fixed and FFA (Plaque assay for SARS‐CoV) was used to detect the neutralization titres.

### Cytokine Secretion by Specific Immune Cells

Single‐cell suspensions were prepared from spleens as previously described. 5 × 10^5^ spleen cells were stimulated with MERS‐CoV, SARS‐CoV, and SARS‐CoV‐2 peptide pool for 24 h at 37 °C. After 24 h of incubation, cytokine secretion was determined by analysing the supernatants with sandwich ELISA kits for IFN‐γ, IL‐2 according to the manufacturer's instructions.

### Binding Assay by ELISA

MERS‐CoV, SARS‐CoV, and SARS‐CoV‐2 RBD‐specific antibodies were provided by researchers working on antibody screening in the same laboratory. For ELISA assay, 96‐well high binding plates were coated with 1 µg ml^−1^ MERS‐CoV, SARS‐CoV, and SARS‐CoV‐2 RBD proteins, and incubated overnight at 4 °C. Washed the plates with PBST three times, the plates were blocked with 10% FBS for 2 h at 37 °C. Then, 100 µl of diluted protein was added to the appropriate wells and incubated at 37 °C for 2 h, followed by an HRP‐conjugated goat anti‐human IgG antibody at 37 °C for 1 h. Then plates were washed seven times, and 100 µl of TMB (3,3,5,5, tetramethylbenzidene) substrate was added to each well and incubated for 5–10 min protected from light. The reaction was stopped with 50 µl of 2 m H_2_SO_4_, and absorbance was measured at a wavelength of 450 nm.

### Binding Assay by BLI

Binding experiments were conducted by saturating captured Mosaic RBD‐np with a 200 nm of MERS‐CoV RBD antibody followed by 100 nm SARS‐CoV‐2 RBD antibody, and 100 nm of SARS‐CoV RBD. The additional resonance response (RU) generated by antibody binding was measured by a FORTEBIO‐OCTET.

### Draining Lymph Nodes Isolation and Flow Cytometry

The draining lymph nodes were dissected from the immunized mice and homogenized through a 70 µm strainer. For analysis of Tfh cells, the cells in the draining lymph nodes were stained at 4 °C for 1 h with a biotin‐CXCR5 (BioLegend), and the following antibodies anti‐PD‐1‐PE‐Cy7, anti‐CD4‐BV421, anti‐CD16/32‐Percp5.5, strep‐PE, the viability maker (FVS 440) were stained at 4 °C for 15 min. Data were analyzed using FlowJo software. For analysis of GC cells, the cells in the draining lymph nodes were stained with the viability maker (FVS 440), anti‐CD45‐APC‐Cy7, anti‐CD19‐PE‐Cy7, anti‐CD38‐BV605, anti‐GL7‐BV421 at 4 °C to acquire the stained cells.

### B Cell Probe Staining

Biotinylated SARS‐CoV‐2 and SARS‐CoV RBDs were produced by co‐transfection of Avi/His‐tagged RBD expression plasmids with an expression plasmid encoding BirA enzyme. RBD proteins were purified from transiently‐transfected Freestyle 293F cell (Gibco) supernatants by nickel affinity and size‐exclusion chromatography. To prepare fluorochrome‐conjugated streptavidin‐tetramerized RBDs, biotinylated SARS‐CoV‐2, and SARS‐CoV RBDs were incubated with streptavidin‐A488 (Invitrogen) and streptavidin‐PE (Invitrogen), respectively, overnight at 4 °C at a 1:4 molar ratios of RBD to streptavidin subunit. For analysis of RBD‐specific B cells, the 1 × 10^6^ cells in the draining lymph nodes were stained at 4 °C for 1 h with 6 µg SARS‐RBD‐A488, 4 µg SARS‐2‐RBD‐PE, and the following antibodies the viability maker (FVS 440), anti‐CD45‐APC‐Cy7, anti‐CD19‐PE‐Cy7, anti‐CD38‐BV605, anti‐GL7‐BV421 at 4 °C for 15 min to acquire the stained cells.

### Quantification and Statistical Analysis

Statistical significance was assigned when *P* values were < 0.05 using Prism Version 9.0 software (GraphPad). Exact *p* values are in the relevant figure near each corresponding line, with asterisks denoting level of significance (^*^ denotes 0.01 < *p* < 0.05, ^**^ denotes 0.001 < *p* < 0.01, ^***^ denotes 0.0001 < *p* < 0.001, and ^****^ denotes *p* < 0.0001). Tests, number of animals (n), median values, and statistical comparison groups are indicated in the Figure legends.

### Ethics Statement

All animal experiments were performed in animal facilities under specific pathogen‐free conditions. The handling of mice and experimental procedures were approved by the Animal Welfare and Research Ethics Committee of the First Affiliated Hospital of Guangzhou Medical University (2020164).

## Conflict of Interest

The authors declare no conflict of interest.

## Supporting information

Supporting Information

## Data Availability

The data that support the findings of this study are available on request from the corresponding author. The data are not publicly available due to privacy or ethical restrictions.
