# Supplementary material for: Mosaic RBD Nanoparticles Elicit Protective Immunity Against Multiple Human Coronaviruses in Animal Models
Source: Adv Sci (Weinh). 2023 Dec 17;11(9):2303366. doi: 10.1002/advs.202303366 (PMC10916629; doi:10.1002/advs.202303366)
Supplement: Supplementary file 1 — Supporting Information [file ADVS-11-2303366-s001.pdf]

ADVANCED SCIENCE
Open Access

Supporting Information

for *Adv. Sci.*, DOI 10.1002/advs.202303366

Mosaic RBD Nanoparticles Elicit Protective Immunity Against Multiple Human Coronaviruses in Animal Models

Yanjun Zhang, Jing Sun, Jian Zheng, Suxiang Li, Haiyue Rao, Jun Dai, Zhaoyong Zhang, Yanqun Wang, Donglan Liu, Zhao Chen, Wei Ran, Airu Zhu, Fang Li, Qihong Yan, Yiliang Wang, Kuai Yu, Shengnan Zhang, Dong Wang, Yanhong Tang, Banghui Liu, Linling Cheng, Jiandong Huo, Stanley Perlman*, Jingxian Zhao* and Jincun Zhao**

Supporting Information

Mosaic RBD nanoparticles elicit protective immunity against multiple human coronaviruses in animal models

Yanjun Zhang [1#], Jing Sun [1#], Jian Zheng [2#], Suxiang Li [1#], Haiyue Rao [1#], Jun Dai [3#], Zhaoyong Zhang [1], Yanqun Wang [1], Donglan Liu [1], Zhao Chen [1], Wei Ran [1], Airu Zhu [1], Fang Li [1], Qihong Yan [1], Yiliang Wang [1], Kuai Yu [1], Shengnan Zhang [1], Dong Wang [1], Yanhong Tang [1], Banghui Liu [4], Linling Cheng [1], Jiandong Huo [1,5*], Stanley Perlman [2*], Jingxian Zhao [1*], Jincun Zhao [1,5,6,7,8*]

[1]State Key Laboratory of Respiratory Disease, National Clinical Research Center for Respiratory Disease, Guangzhou Institute of Respiratory Health, the First Affiliated Hospital of Guangzhou Medical University, Guangzhou, China.

[2]Department of Microbiology and Immunology, University of Iowa, Iowa City, Iowa 52242, USA.

[3]Guangzhou Customs District Technology Center, Guangzhou 510700, China.

[4]State Key Laboratory of Respiratory Disease, Guangdong Laboratory of Computational Biomedicine, Guangzhou Institutes of Biomedicine and Health, Chinese Academy of Sciences, Guangzhou, People's Republic of China.

[5]Guangzhou laboratory, Bio-island, Guangzhou, China.

[6]Institute of Infectious disease, Guangzhou Eighth People's Hospital of Guangzhou Medical University, Guangzhou, China.

[7]Institute for Hepatology, National Clinical Research Center for Infectious Disease, Shenzhen Third People’s Hospital, the Second Affiliated Hospital, School of Medicine, Southern University of Science and Technology, Shenzhen, China.

[8]Shanghai Institute for Advanced Immunochemical Studies, School of Life Science and Technology, ShanghaiTech University, Shanghai, 201210, China.

[#]Co-first author, these authors contributed equally to this work.

*Corresponding authors.

Jincun Zhao, State Key Laboratory of Respiratory Disease, National Clinical Research Center for Respiratory Disease, Guangzhou Institute of Respiratory Health, the First Affiliated Hospital of Guangzhou Medical University, Guangzhou, China, email: zhaojincun@gird.cn

Jingxian Zhao, State Key Laboratory of Respiratory Disease, National Clinical Research Center for Respiratory Disease, Guangzhou Institute of Respiratory Health, the First Affiliated Hospital of Guangzhou Medical University, Guangzhou, China, email: zhaojingxian@gird.cn

Stanley Perlman Department of Microbiology and Immunology, University of Iowa, Iowa City, Iowa 52242, USA; Department of Pediatrics, University of Iowa, Iowa City, Iowa 52242, USA. Electronic address: stanley-perlman@uiowa.edu

Jiandong Huo, State Key Laboratory of Respiratory Disease, National Clinical Research Center for Respiratory Disease, Guangzhou Institute of Respiratory Health, the First Affiliated Hospital of Guangzhou Medical University, Guangzhou, China, email: huojiandong@gird.cn

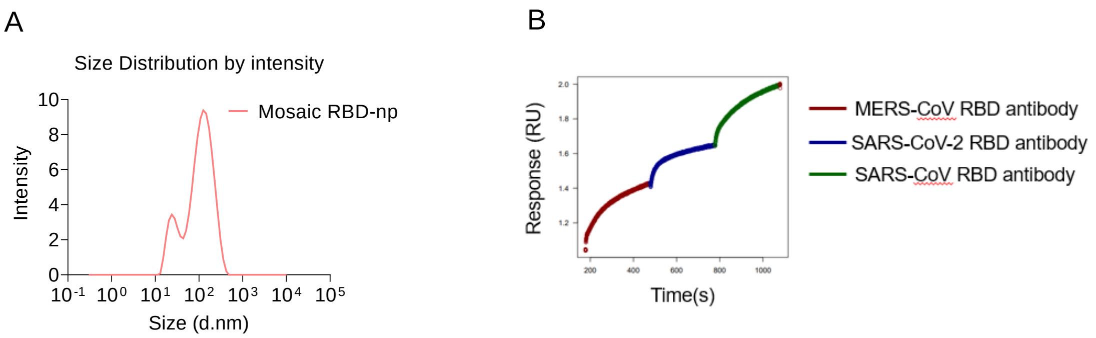

Fig. S1. Related to Fig.1. Construction and characterization of mosaic-RBD nanoparticles. (A) DLS analysis in intensity for the Mosaic RBD-np. (B) the binding assay of Mosaic-RBD nanoparticles with the SARS-CoV-2, SARS-CoV and MERS-CoV RBD-specific monoclonal antibodies by BLI.

SARS-CoV-2 RBD-Ferritin>

SFTVEKGIYQTSNFRVQPTESIVRFPNITNLCPFGEVFNATRFASVYAWNRKRISNCVADYSVLY
NSASFSTFKCYGVSPTKLNDLCFTNVYADSFVIRGDEVRQIAPGQTGKIADYNYKLPDDFTGCVI
AWNSNNLDSKVGGNYNYLYRLFRKSNLKPFERDISTEIYQAGSTPCNGVEGFNCYFPLQSYGF
QPTNGVGYQPYRVVVLSFELLHAPATVCGPKKSSGGGSGGGESQVRQQFSKDIIKLLNEQVNK
EMQSSNLYMSMSSWCYTHSLDGAGLFLFDHAAEEYEHAKKLIIFLNENNVPVQLTSISAPEHKF
EGLTQIFQKAYEHEQHISESINNIVDHAIKSKDHATFNFLQWYVAEQHEEEVLFKDILDKIELIGNE
NHGLYLADQYVKGIAKSRKS

SARS-CoV RBD-Ferritin>

NITNLCPFGEVFNATKFPSVYAWERKKISNCVADYSVLYNSTFFSTFKCYGVSATKLNDLCFSNV
YADSFVVKGDDVRQIAPGQTGVIADYNYKLPDDFMGCVLAWNTRNIDATSTGNYNYKYRYLRH
GKLRPFERDISNVPFSPDGKPCTPPALNCYWPLNDYGFYTTTGIGYQPYRVVVLSFELLNAPAT
VSGGGSGGGESQVRQQFSKDIIKLLNEQVNKEMQSSNLYMSMSSWCYTHSLDGAGLFLFDHA
AEEYEHAKKLIIFLNENNVPVQLTSISAPEHKFEGLTQIFQKAYEHEQHISESINNIVDHAIKSKDH
ATFNFLQWYVAEQHEEEVLFKDILDKIELIGNENHGLYLADQYVKGIAKSRKS

MERS-CoV RBD-Ferritin>

QAEGVECDFSPLLSGTPPQVYNFKRLVFTNCNYNLTKLLSLFSVNDFTCSQISPAAIASNCYSSLI
LDYFSYPLSMKSDLSVSSAGPISQFNYKQSFSNPTCLILATVPHNLTTITKPLKYSYINKCSRLLS
DDRTEVPQLVNANQYSPCVSIVPSTVWEDGDYYRKQLSPLEGGGWLVASGSTVAMTEQLQMG
FGITVQYGTDTNSVCPKLSGGGSGGGESQVRQQFSKDIIKLLNEQVNKEMQSSNLYMSMSSW
CYTHSLDGAGLFLFDHAAEEYEHAKKLIIFLNENNVPVQLTSISAPEHKFEGLTQIFQKAYEHEQH
ISESINNIVDHAIKSKDHATFNFLQWYVAEQHEEEVLFKDILDKIELIGNENHGLYLADQYVKGIAK
SRKS

Table S1. Amino acid sequences for nanoparticles used in this study.

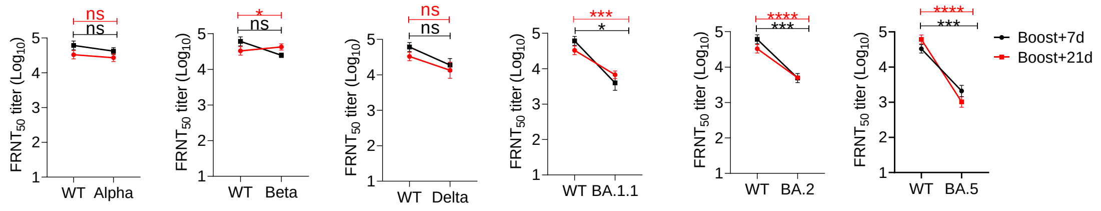

Fig. S2. Comparison of the neutralization titres against SARS-CoV-2 WT strain and its variants. (A) Red and dark line represent the day 7 and day 21 after the boost immunization. All results are expressed as mean ± SEM. Statistical analyses were performed using two-way ANOVA. (B) Realted information about Fig.2.D-F (C) Divergence amino acids in the RBD domain of MERS-CoV Strains used in this study.

A

	Mean Neutralization titer (boost+7d)	Neutralization assay	Number of mice in this experiment
MERS-CoV EMC	3.34 (Log10)	Focus formation assay	3
MERS-CoV GD01	3.39 (Log_{10})	Pseudovirus assay	3
MERS-CoV Nigeria	3.30 (Log_{10})	Pseudovirus assay	3
SARS-CoV	3.80 (Log_{10})	Plaque formation assay	5
SARS-CoV-2 WT	4.52 (Log_{10})	Focus formation assay	3
SARS-CoV-2 Alpha	4.43 (Log_{10})	Focus formation assay	4
SARS-CoV-2 Beta	4.63 (Log_{10})	Focus formation assay	4
SARS-CoV-2 Delta	4.13 (Log_{10})	Focus formation assay	4
SARS-CoV-2 BA.1	3.83 (Log_{10})	Focus formation assay	4
SARS-CoV-2 BA.2	3.70 (Log_{10})	Focus formation assay	5
SARS-CoV-2 BA.5	3.32 (Log_{10})	Focus formation assay	6

B

MERS-CoV strains	Divergence in the RBD
MERS-CoV EMC/2012	L495, L588
MERS-CoV ChinaGD01	L495, L588
MERS-CoV Nigeria (Nig1675)	F495, F588

Table S2. (A) relevant information about Fig. 2. **(B)** the RBD sequence divergence of MERS CoV EMC, GD01 and Nigeria.

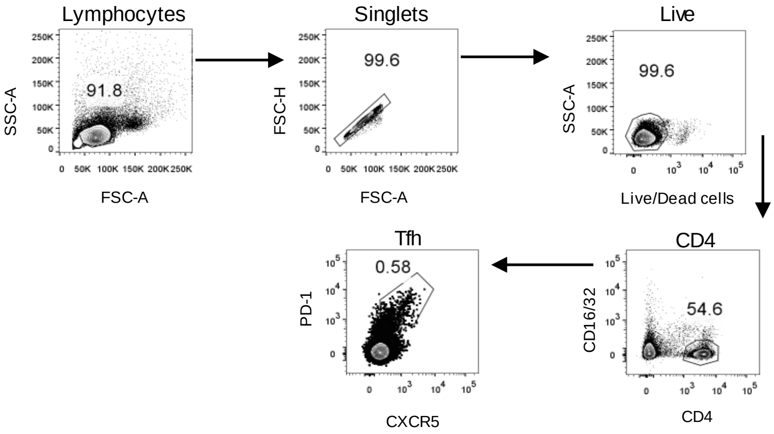

B GC B cells

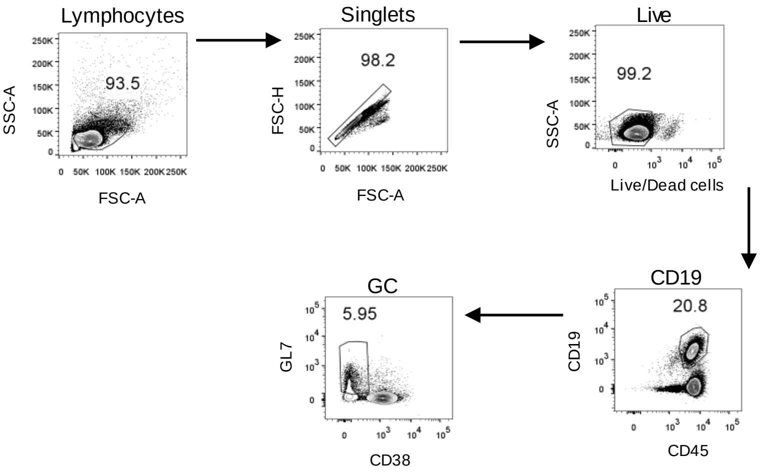

Fig. S3. Tfh and B cell gating strategy. Cells were gated as singlets and live cells on forward and side scatter and a live/dead FVS440 stain. (**A**) CD16/32 negative, CD4 positive cells were then gated on the expression of CXCR5 and PD-1. (**B**) CD45 and CD19 positive cells were gated, and then GC B cells were selected based on lower expression of CD38 and positive expression of GL7.

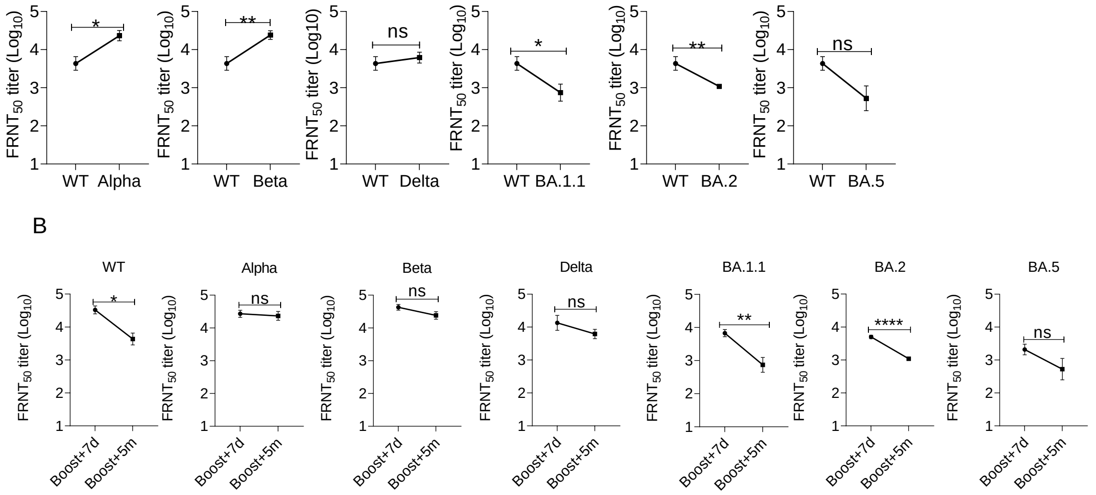

Fig. S4. Comparison of neutralization titres. (**A**) Comparison of neutralization titres between SARS-CoV-2 WT strain and different variants. (**B**) Comparison of neutralization titres between 7 days and 5 months after booster immunization for different strains. All results are expressed as mean ± SEM. Statistical analyses were performed using unpaired t test.

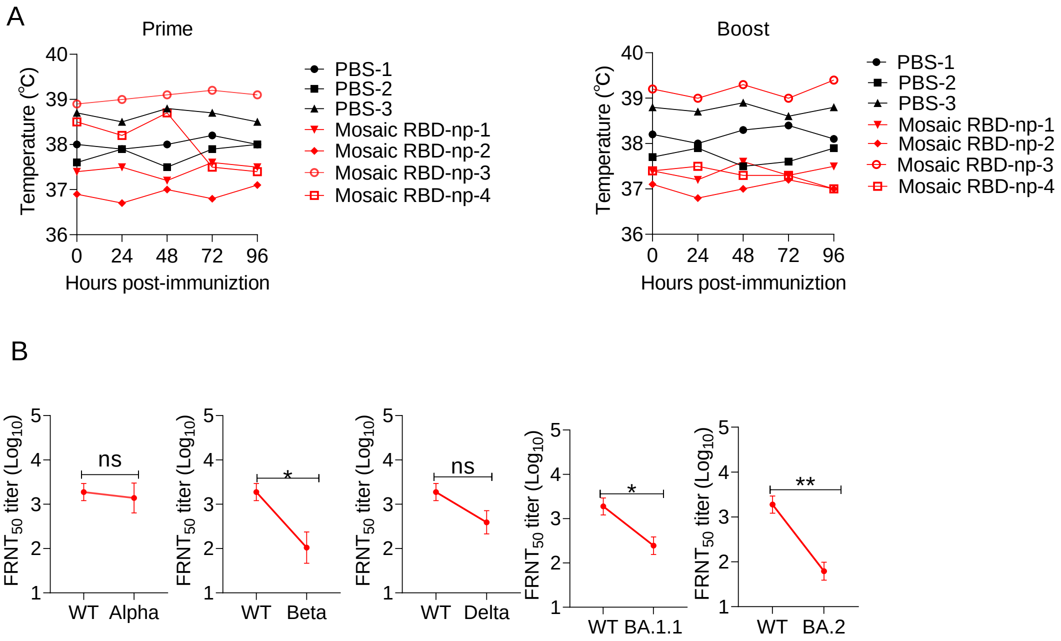

Fig. S5. Temperature change after immunization of RBD-np vaccine in cynomolgus monkeys and comparison of neutralization titres induced by immunization. (**A**) Temperature was monitored within 96 hours of primary and booster immunizations. (**B**) Comparison of the neutralization titres against SARS -CoV-2 WT strain and its variants. All results are expressed as mean ± SEM. Statistical analyses were performed using unpaired t test.

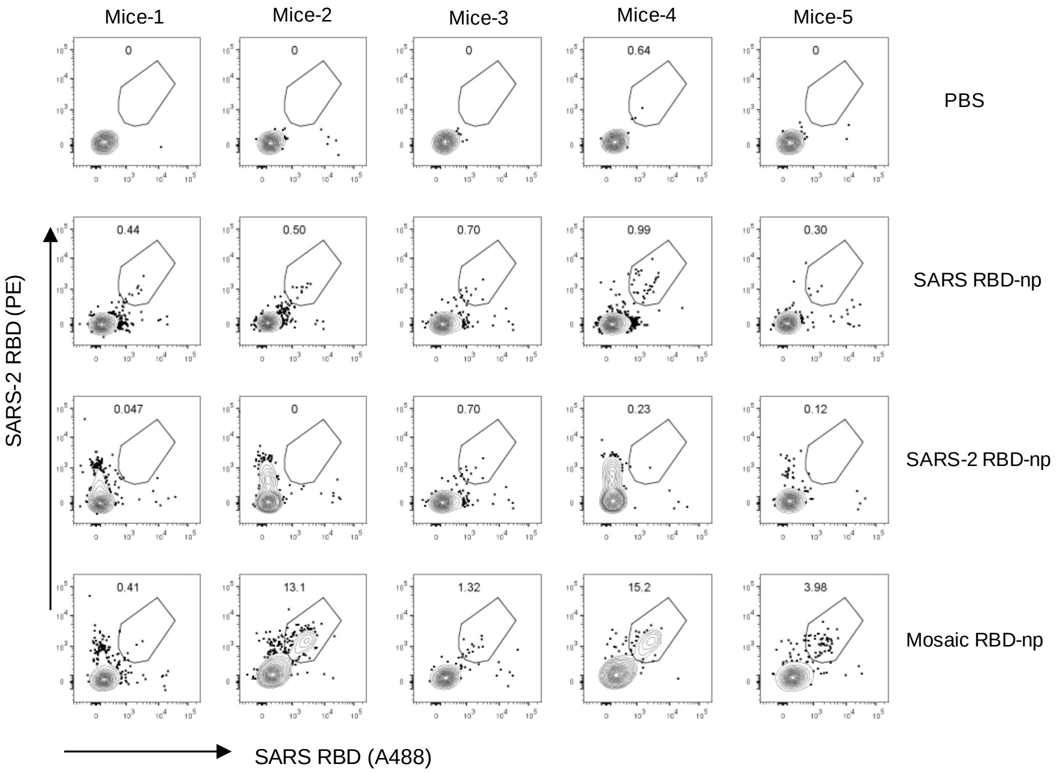

Fig. S6. Complete flow cytometry analysis for cross reactive-RBD⁺ GC B cells isolated from mice immunized with heterotypic mosaic-RBD nanoparticles or homotypic RBD nanoparticles (SARS-CoV RBD-np and SARS-CoV-2 RBD-np).
